# Existence of common fuzzy fixed points via fuzzy *F*-contractions in b-metric spaces

**DOI:** 10.1038/s41598-024-58451-7

**Published:** 2024-04-02

**Authors:** Shazia Kanwal, Sana Waheed, Ariana Abdul Rahimzai, Ilyas Khan

**Affiliations:** 1 Department of Mathematics, Government College University, Faisalabad, Pakistan; 2 Department of Mathematics, Education Faculty, Laghman University, Mehtarlam, Laghman 2701 Afghanistan; 3 Department of Mathematics, Saveetha School of Engineering, SIMATS, Chennai, Tamil Nadu India; 4 Department of Mathematics, College of Science Al-Zulfi Majmaah University, Al-Majmaah, 11952 Saudi Arabia

**Keywords:** Fuzzy set, fuzzy mapping, *b*-metric, Hausdorff metric, *F*-contraction, Mathematics and computing, Statistical physics, thermodynamics and nonlinear dynamics

## Abstract

The main goal of this study is to establish common fuzzy fixed points in the context of complete b-metric spaces for a pair of fuzzy mappings that satisfy F-contractions. To strengthen the validity of the derived results, non-trivial examples are provided to substantiate the conclusions. Moreover, prior discoveries have been drawn as logical extensions from pertinent literature. Our findings are further reinforced and integrated by the numerous implications that this technique has in the literature. Using fixed point techniques to approximate the solutions of differential and integral equations is very useful. Specifically, in order to enhance the validity of our findings, the existence result of the system of non-linear Fredholm integral equations of second-kind is incorporated as an application.

## Introduction

Initiation of fuzzy set theory in 1965 by Zadeh^1^ helps us to make possible the description of vague notions and handling with them. Basically, a fuzzy set is a function whose domain is a non-empty set and range is the interval [0,1]. At first, Weiss^2^ and Butnariu^3^ studied fixed points of fuzzy mappings. The concept of fuzzy contraction mappings was introduced by Heilpern^4^ (see also^5,6^). Afterwards, existence of fixed points of mappings involving certain contractive type conditions were derived and calculated by several authors, for example fuzzy common fixed points of fuzzy mappings for integral type contractions were obtained by Kanwal et al.^7^, fuzzy fixed points and common fixed points were established by Azam et al.^8,9^. Further, fuzzy fixed point results involving Nadler’s type contractions were established by Kanwal et al.^10,11^. With the aim of generalization of the Banach contraction principle, instead of the triangle inequality, a weaker condition was used in this metric space, and these spaces are known as *b*-metric spaces. The idea of a *b*-metric space was first presented by Backhtin^12^ in 1989. Czerwik^13^ extracted the *b*-metric space results in 1993. Many scholars generalized the Banach contractive principle in *b*-metric spaces by embracing this theory. The existence of fixed points and common fixed points of fuzzy mappings satisfying the contractive type criterion is deduced and estimated by several authors. Fixed point theorems in *b*-metric spaces were obtained by Boriceanu^14^, Czerwik^13^, Kir and Kiziltunc^15^, Kumam et al.^16^, Kanwal et al.^17^ and Pacurar^18^. Fuzzy fixed point theorems for multivalued fuzzy contractions in *b*-metric spaces were proved by Phiangsungnoen and Kumam^19^. In past few decades, a noteworthy interest in fixed point theory has been directed to interchanging recent metric fixed point results from usual metric spaces to some other metric spaces, like quasi-metric spaces, partially ordered metric spaces, psuedo metric spaces, *F*-metric spaces, rectangular metric spaces, fuzzy metric spaces, etc. Nadler^20^ extended the Banach contraction principle^21^ and obtained the fundamental fixed point result for set valued mappings using the Hausdroff metric. These non-linear diversity Problems open the door to develop more original and innovative tools, which are currently receiving more attention in literature. Wardowski^22^ used one of these tools, which is thought to be a novel tool, in which the author introduced a new type of contractions, called $\texttt {F}$-contractions and proved a new related fixed point theorem.

Some fuzzy fixed point theorems for fuzzy mappings via $\texttt {F}$-contractions were shown by Ahmad et al.^23^. Several other authors have studied and obtained fixed point theorems for $\texttt {F}$-contractions (see^24,25^ and references therein). A survey on F-contraction can be obtained from^26^. Recently, Kanwal et al.^17^ obtained common fixed points of *L*-fuzzy mappings satisfying $\texttt {F}$-contractions in complete *b*-metric spaces. Dhanraj et al.^27^, Gopal et al.^28,29^, Lakzian et al.^30^, Mani et al.^31,32^ and Nallaselli et al.^33^ have established many wonderful results in b-metric spaces and its generalizations for F- contractions and some other contractive conditions. Moreover, they have offered the applications of the obtained results, see references therein for details.

The purpose of this study is to obtain common fuzzy fixed points of two fuzzy mappings in the setting of complete *b*-metric spaces via fuzzy $\texttt {F}$-contractions in connection with Housdorff metric. The structure of the paper is as follows: “Preliminaries” section deals with basic concepts regarding definitions, examples and lemmas which are necessary to understand our results. Common fuzzy fixed point results via $\texttt {F}$-contractions in complete b-metric spaces with consequences and interesting examples have been given in “Common fuzzy fixed points via *F*-contraction” section. In “Applications” section, we have established common fixed points for multivalued mappings and solve a non-linear system of Fredhlem integral equations of 2nd kind by our findings. A conclusion is incorporated in “Conclusion” section.

## Preliminaries

In this section some pertinent concepts are presented from the existing literature. These concepts will be helpful to understand the results which are established in the present research.

### **Definition 2.1**

Let (*S*, *d*) be a metric space and $S^{CB}$ denotes the collection of all nonempty closed and bounded subsets of *S*. Consider a map $H:S^{CB}\times S^{CB} \rightarrow \mathbb {R}.$ For $\theta ,\vartheta \in S^{CB}$ defined by

$$\begin{aligned} H(\theta ,\vartheta )= \max \{\sup _{c\in \theta }d(c,\vartheta ), \sup _{e\in \vartheta }d(e,\theta )\}, \end{aligned}$$

where $d(c,\vartheta )=\{\inf d(c,e): e \in \vartheta \}$ is the distance of *c* to $\vartheta .$ *H* is a metric on $S^{CB}$ and is known as the Hausdorff metric induced by the metric *d*.

### **Definition 2.2**

Let (*W*, *d*) be a metric space. A mapping $\Gamma : W \rightarrow W$ is Banach contraction on W if there exists a positive real number $0< \gamma < 1$ such that $\forall z_{1}, z_{2} \in W$,

$$\begin{aligned} d(\Gamma z_{1},\Gamma z{2}) \le \gamma d(z_{1}, z_{2}). \end{aligned}$$

### **Definition 2.3**

A mapping $\Gamma$ defined on metric space (*W*, *d*) satisfying

$$\begin{aligned} d(\Gamma v, \Gamma w) \le \gamma [d(v, \Gamma v)+ d(w, \Gamma w)] \ \ \ \ \ \forall v, w \in W, \end{aligned}$$

where $\gamma \in [0, \frac{1}{2})$ is called Kannan contraction.

### **Definition 2.4**

^1^ Let *S* be any arbitrary set. A function $\mu :S \rightarrow [0,1]$ is called a fuzzy set in *S*. The functional value $\mu (s)$ is called the grade of membership of *s* in $\mu .$ The $\alpha$-level set of $\mu$ is denoted by $[\mu ]_{\alpha }$ and is defined as follows:

$$\begin{aligned}{}[\mu ]_{\alpha }=\{s; \mu (s)\ge \alpha \,\, if \,\, \alpha \in (0,1]\}. \end{aligned}$$

**Note:** Throughout the article, we denote $S^*$ as the family of fuzzy sets in *S*, $S^{CB}$ denotes the collection of all closed and bounded subsets of *S* and *C*(*S*) denotes the collection of all compact sets.

### *Example 2.5*

Consider functions $A_{1}, \ A_{2}$ on [0, 70] defined by: $$ A_{1}(x)=\left\{ \begin{array}{ll} 1, &{} \hbox {when }\, x \le 20; \\ {\frac{7}{3}-\frac{x}{15}}, &{} \hbox {when }\,20< x < 35; \\ 0, &{} \hbox {when } x \ge 35. \end{array} \right. $$

$$ A_{2}(x)=\left\{ \begin{array}{ll} 0, &{} \hbox {when either }\,x \le 20 \,\textrm{or}\, \ge 60; \\ \frac{(x-20)}{15}, &{} \hbox {when }\, 20< x<35; \\ \frac{(60-x)}{15}, &{} \hbox {when}\, 45< x <60; \\ 1, &{} \hbox {when}\, 35\le x \le 45. \end{array} \right. $$

**Figure 1:**
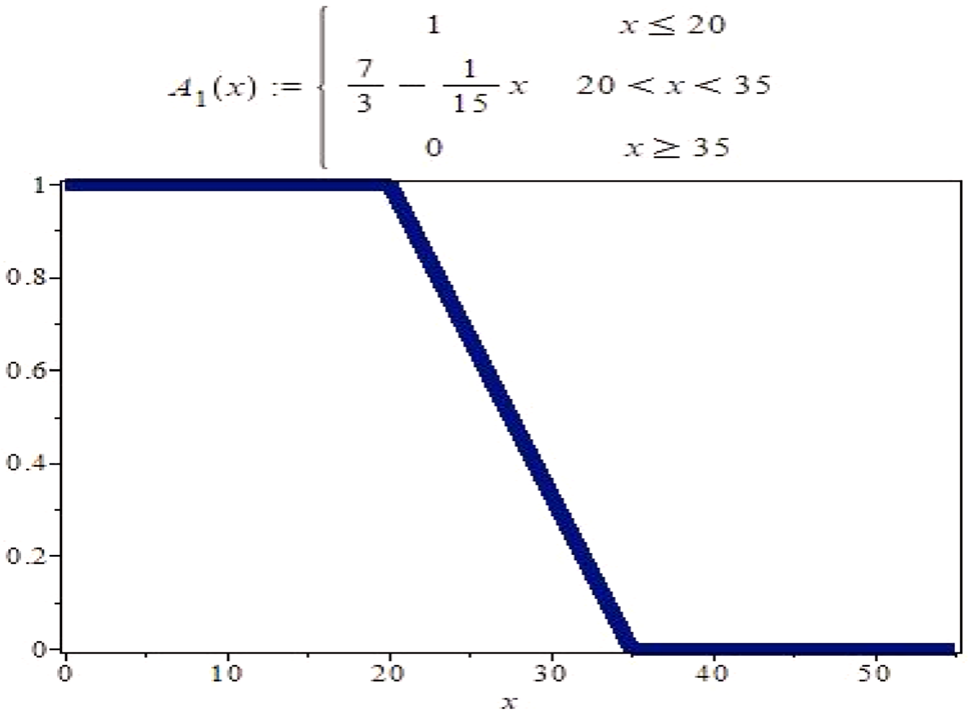
Graph of fuzzy set $A_{1}$.

**Figure 2:**
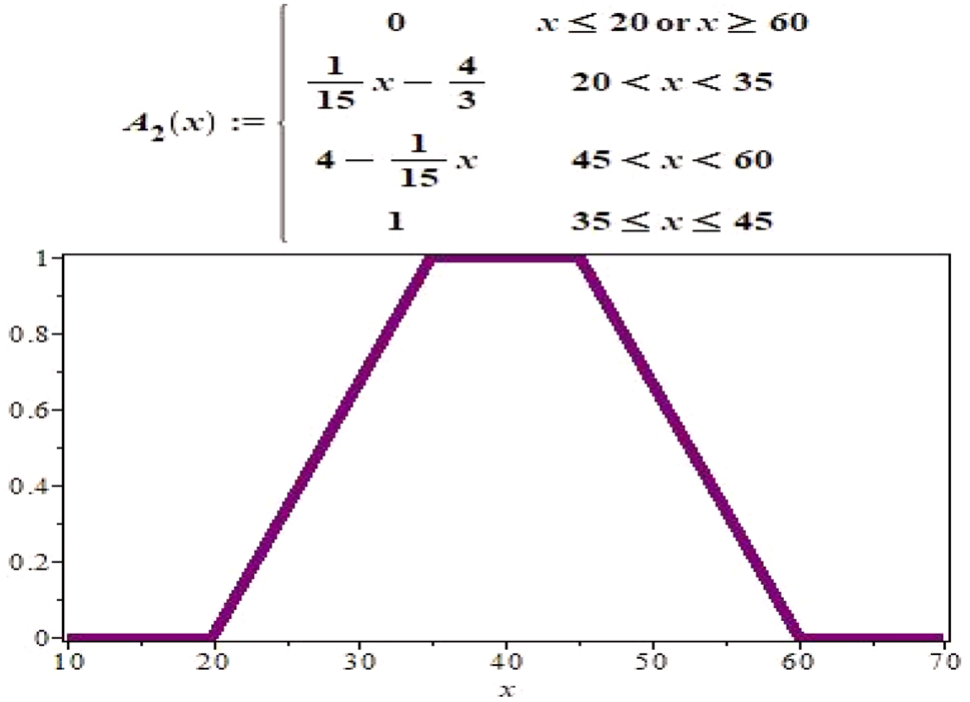
Graph of fuzzy set $A_{2}$.

Both $A_{1}, \ A_{2}$ are fuzzy sets. Graphical representation of $A_{1}$ and $\ A_{2}$ can be seen in Figs. 1 and 2, respectively.

### **Definition 2.6**

^4^ Let (*S*, *d*) be any metric space and *P* be an arbitrary set. *T* is termed as a fuzzy mapping if $T: P \rightarrow S^{*}$ is a function i.e $T(p) \in S^{*}$ for each $p \in P.$

### *Example 2.7*

Let $P= [-9,9]$ and $S=[-4, 4]$. Define $T_{1}:P \longrightarrow S^{*}$ by

$$\begin{aligned} T_{1}(x)(y)= \frac{x^{2} + y^{2} }{100}. \end{aligned}$$

**Figure 3:**
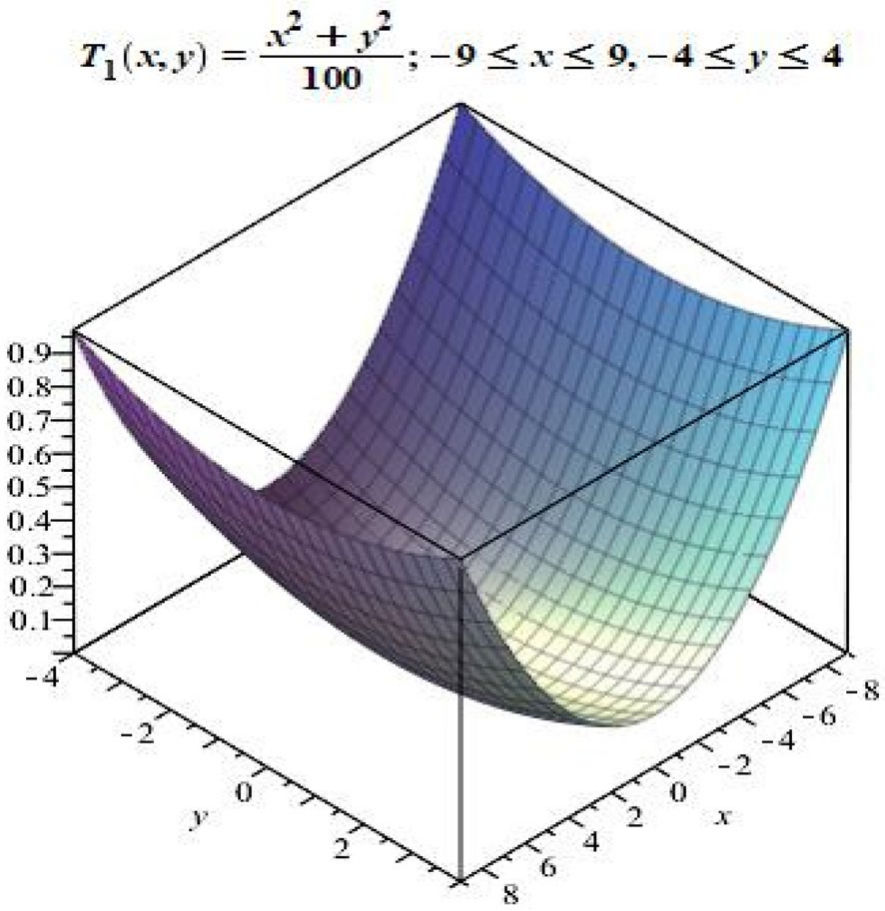
Graph of fuzzy mapping $T_{1}$.

Then $T_{1}$ is a fuzzy mapping. Notice that $T_{1}(x)(y)\in [0,1]$, for all $x \in P$ and $y\in S$. The graphical representation $T_{1}(x)(y)$ showing the possible membership values of *y* in $T_{1}(x)$ is given in Fig. 3.

### *Example 2.8*

Let $P= [0,15]$ and $S=[0, 10]$. Define $T_{2}:P \longrightarrow S^{*}$ by

$$\begin{aligned} T_{2}(x)(y)= \frac{x + y +xy }{180}. \end{aligned}$$

**Figure 4:**
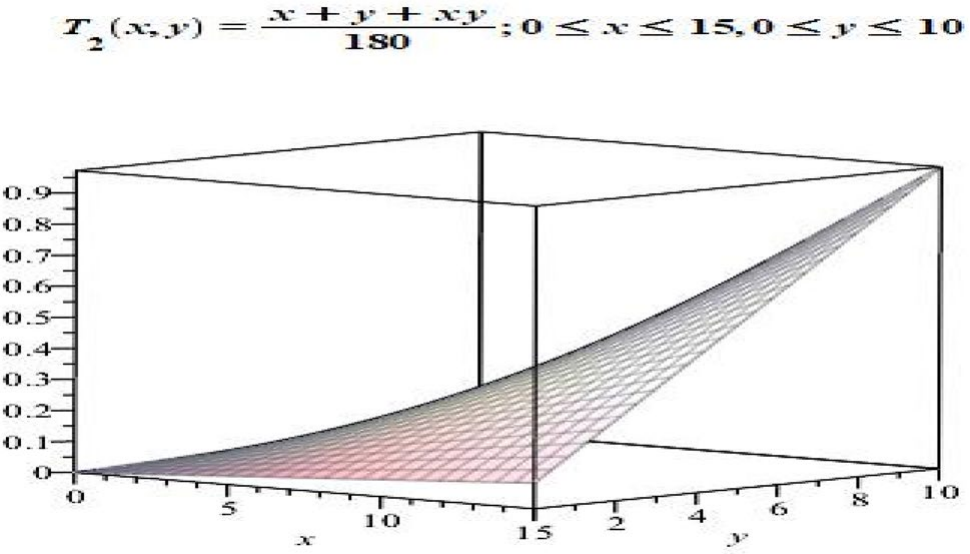
Graph of fuzzy mapping $T_{2}$.

Then $T_{2}$ is a fuzzy mapping. Notice that $T_{2}(x)(y)\in [0,1]$, for all $x \in P$ and $y\in S$. The graphical representation $T_{2}(x)(y)$ showing the possible membership values of *y* in $T_{2}(x)$ is given in Fig. 4.

### **Definition 2.9**

Let $G, T:S \longrightarrow S^{*}$ be fuzzy mappings. An element $u\in S$ is called a fuzzy fixed point of *G* if $u\in [Gu]_\alpha$. The point *u* is called a common fuzzy fixed point of *G* and *T* if $u\in [Gu]_\alpha \cap [Tu]_\alpha$.

### **Definition 2.10**

^12^ Consider *S* to be a non-empty set and $y\ge 1$. Assume the function $d^{*}: S\times S \rightarrow \mathbb {R}$ satisfies the following conditions for all $\xi _{1},\xi _{2},\xi _{3} \in S$:

- $$d^{*}(\xi _{1}, \xi _{2})= 0, \ \Rightarrow \, \xi _{1}=\xi _{2};$$
- $$d^{*}(\xi _{1}, \xi _{2}) > 0$$ for all $$\ \xi _{1}\ne \xi _{2}$$
- $$d^{*}(\xi _{1}, \xi _{2})= d^{*}(\xi _{2},\xi _{1});$$
- $$d^{*}(\xi _{1}, \xi _{2})\le y(d^{*}(\xi _{1}, \xi _{3})+ d^{*}(\xi _{3}, \xi _{2})).$$

Then $d^{*}$ is a *b*-metric on *S* and the pair $(S,d^{*},y)$ is referred as a *b*-metric space.

### *Example 2.11*

^14^ The space $$l_{\zeta } (0< \zeta < 1)$$,

$$\begin{aligned} l_{\zeta } = \{(\mu _{n}) \subset {\mathbb {R}}: \sum _{n=1}^{\infty } |\mu _{n}|^{\zeta } < \infty \}, \end{aligned}$$

together with a function $d^{*}: l_{\zeta }\times l_{\zeta } \rightarrow {\mathbb {R}}$

$$\begin{aligned} d^{*}(\mu ,\nu ) = \big ( \sum _{n=1}^{\infty } |\mu _{n}-\nu _{n}|^{\zeta }\big )^{\frac{1}{\zeta }}, \end{aligned}$$

where $\mu =(\mu _{n}),\ \nu =(\nu _{n}) \in l_{\zeta }$ is a b-metric space. By an elementry calculation we obtain that

$$\begin{aligned} d^{*}(\mu ,z) \le 2^{\frac{1}{\zeta }}\big [d^{*}(\mu ,\nu ) + d^{*}(\nu ,z)\big ]. \end{aligned}$$

Here $$s=2^{\frac{1}{\zeta }}>1$$.

### *Example 2.12*

^14^ The space $L_{\zeta } (0< \zeta < 1)$, of all real functions $\mu (t), t \in [0,1]$ such that $\int _{0}^{1} |\mu (t)|^{\zeta } dt < \infty ,$ is b-metric space if we take

$$\begin{aligned} d^{*}(\mu ,\nu ) = \big [\int _{0}^{1} |\mu (t)-\nu (t)|^{\zeta } dt\big ]^{\frac{1}{\zeta }}, \end{aligned}$$

for each $$\mu ,\nu \in L_{\zeta }$$.

**Remark:** Note that a (usual) metric space is evidently a b-metric space. However Czerwik^13^ has shown that a b-metric on *X* need not be a metric on *X*.

### **Definition 2.13**

Let $(S,d^{*},y)$ be a *b*-metric space with $y\ge 1.$ Assume $\{s_{n}\}$ is a sequence in *S* and $s \in S$. *s* is termed as the limit of the sequence $\{s_{n}\}$ if

$$\begin{aligned} \lim _{n \rightarrow \infty } d^{*}(s_{n},s)=0. \end{aligned}$$

Then $\{s_{n}\}$ is said to be convergent in *S*.

### **Definition 2.14**

The sequence $$\{s_{n}\}$$ in the *b*-metric space $$(S,d^{*},y)$$ is said to be Cauchy if for each $$\epsilon > 0$$, there is $$ n_{0}$$ a positive integer such that $$d^{*}(s_{n},s_{m})< \epsilon $$ for all $$n,m > n_{0}.$$

### **Definition 2.15**

If every Cauchy sequence in $(S,d^{*},y)$ is convergent in *S*, thenthe *b*-metric space $(S,d^{*},y)$ is said to be complete.

### **Definition 2.16**

^34^ Let $y\ge 1$ be a real number. Suppose that $\textsf {F}^*$ is the family of all functions $\texttt {F} : \mathbb {R^{+}} \rightarrow \mathbb {R}$ satisfying the conditions given below:

$$(F_{1})$$
$\texttt {F}$ is strictly increasing;
$$(F_{2})$$
for each positive sequence $\{s_{n}\}$, $\lim _{n \rightarrow \infty } s_{n}=0 \,\Leftrightarrow \, \lim _{n \rightarrow \infty } \texttt {F}(s_{n})=-\infty ;$
$$(F_{3})$$
There is $k \in (0,1)$ so that $\lim _{n \rightarrow \infty }(s_{n})^{k}{} \texttt {F}(s_{n})=0$ for each $\{s_{n}\}\subset \mathbb {R^{+}};$
$$(F_{4})$$
$\sigma + \texttt {F} (y^{n}s_{n})\le \texttt {F} (y^{n-1}s_{n-1})$ if for $\{s_{n}\}\subset \mathbb {R^{+}}$ and $\sigma \in \mathbb {R^{+}}, \, \sigma + \texttt {F} (ys_{n})\le \texttt {F} (s_{n-1}) \, \forall \, n \in \mathbb {N}.$

### **Definition 2.17**

Let $(S,d^{*},y)$ be a *b*-metric space, where $y\ge 1.$ A multivalued mapping $T:S\rightarrow S^{CB}$ is called an $\texttt {F}$ -contraction of Nadler type if there exists $\texttt {F} \in \textsf {F}^*$ such that for $a \in \mathbb {R}^{+}$ and $s,u\in S$,

1 $$\begin{aligned} 2a+\texttt {F}(yH(Ts,Lu))\le \texttt {F}(\beta \psi (s,u)), \end{aligned}$$

where $$0<\beta <1,$$

2 $$\begin{aligned} \psi (u,s)= \max \{d^{*}(u,s),d^{*}(u,Tu),d^{*}(s,Ls),\frac{d^{*}(u,Ls)+d^{*}(s,Tu)}{2y}\}. \end{aligned}$$

Now, we give some definitions and lemmas about multivalued mappings.

### **Lemma 2.18**

^35,36^ Suppose that $(S,d^{*},y)$ is a *b*-metric space. Assume $C_{1},C_{2},C_{3} \in S^{CB}$ and $s,u \in S$, then the following axioms hold:

1. $$d^{*}(s, C_{2})\le d^{*}(s, c_{2})$$ for any $$c_{2}\in C_{2}$$;
2. $$d^{*}(C_{1}, C_{2})\le H(C_{1},C_{2})$$, where $$d^{*}(C_{1},C_{2})=\inf \{d^{*}(c_{1},c_{2}): c_{1}\in C_{1}$$ and $$c_{2}\in C_{2}\}$$;
3. $$d^{*}(c_{1}, C_{2}) \le H(C_{1}, C_{2})$$ for any $$c_{1}\in C_{1}$$;
4. $$H(C_{1}, C_{1})=0$$;
5. $$H(C_{1}, C_{2})=H(C_{2}, C_{1})$$;
6. $$H(C_{1}, C_{3})\le y[H(C_{1}, C_{2})+H(C_{2}, C_{3})]$$;
7. $$H(c_{1}, C_{1})\le y[d^{*}(c_{1}, c_{2})+d^{*}(c_{2}, C_{1})]$$.

### **Lemma 2.19**

^20^ Suppose $(S,d^{*})$ is a metric space and $C_{1},C_{2}\in S^{CB}$, then for $\beta > 1$ and each $c_{1}\in C_{1}$, there exists $c_{2}(c_{1})\in C_{2}$ such that $d^{*}(c_{1},c_{2}) \le \beta H(C_{1},C_{2}).$

### **Lemma 2.20**

^20^ Suppose $(S,d^{*})$ is a metric space and $C_{1},C_{2}\in S^{CB}$, then for $\beta \ge 1$, for each $c_{1}\in C_{1}$ there exists $c_{2}(c_{1})\in C_{2}$ such that $d^{*}(c_{1},c_{2}) \le \beta H(C_{1},C_{2}).$

Lemma 2.20 has the following implications.

### **Lemma 2.21**

^20^ Suppose $C_{1}$ and $C_{2}$ are two arbitrary non-empty compact subsets of a metric space $(S,d^{*})$ and let $\varpi : C_{1} \rightarrow S^{CB}$ be a multivalued map. Then for $\beta \ge 1$, for each $c_{1},c_{2}\in C_{1}$ and $s \in \varpi c_{1}$ there exists $u\in \varpi c_{2}$ such that $d^{*}(s,u) \le \beta H(\varpi c_{1},\varpi c_{2}).$

### **Lemma 2.22**

^35,36^ Suppose $(S,d^{*},y)$ is a *b*-metric space and $C_{1},C_{2}\in S^{CB}$ then for $\beta > 1$, for each $c_{1}\in C_{1}$ there exists $c_{2}(c_{1})\in C_{2}$ such that $d^{*}(c_{1},c_{2}) \le \beta H(C_{1},C_{2}).$

### **Lemma 2.23**

^37^ Let $\{C_{n}\}$ be a sequence in $S^{CB}$ and $\lim _{n \rightarrow \infty }H(C_{n},C_{1})=0$ for $C_{1}\in S^{CB}$ if $c_{n}\in C_{n}$ and $\lim _{n \rightarrow \infty }d^{*}(c_{n},c_{1})=0$, then $c_{1}\in C_{1}.$

### **Theorem 2.24**

^22^ Let$(\varpi ,m)$ be a complete metric space and $\beta : \varpi \rightarrow \varpi$ be an $\texttt {F}$-contraction. Then, $\beta$ admits a unique fixed point in $\varpi$ and for each $x \in \varpi$, the sequence $\{\beta ^{n} (x_{0})\}$ converges to *x*.

### **Theorem 2.25**

Assume a *b*-metric space $(S,d^{*},y)$, where $y\ge 1.$ Let $T:S\rightarrow S^{CB}$ be an $\texttt {F}$-contraction of Nadler type, that is, there is $\texttt {F} \in \textsf {F}^*$, so that for $a\in \mathbb {R}^{+}$,

3 $$\begin{aligned} 2a+\texttt {F}(yH(Ts_{1},Ls_{2}))\le \texttt {F}(d^{*}(s_{1},s_{2}) \end{aligned}$$

for all $s_{1},s_{2} \in S$ and $Ts_{1}\ne Ts_{2}$. Then *T* admits a fixed point in *S*.

## Common fuzzy fixed points via *F*-contraction

In this section, we have established Nadler’s type common fixed points of a pair of fuzzy-mappings satisfying *F*- contractions in the context of complete *b*-metric space. Examples furnish legitimacy for the conclusions. As corollaries from the pertinent literature, there are also previous conclusions that have been stated.

### **Definition 3.1**

Consider a *b*-metric space $(S,d^{*},y)$, where $y\ge 1.$ Two fuzzy mappings $T, L:S\rightarrow S^*$ is termed as an $\texttt {F}$-contraction of Nadler type if there is $\texttt {F} \in \textsf {F}^*$ so that for $a \in \mathbb {R}^{+}$ and for all $s,u\in S$,

4 $$\begin{aligned} 2a+\texttt {F}(yH([Ts]_{\alpha _{Ts}},[Lu]_{\alpha _{Lu}}))\le \texttt {F}(\beta \psi (s,u)), \end{aligned}$$

where $$y\ge 1, \,\, 0<\beta <1$$, $$\alpha _{Lu}, \ \alpha _{Ts} \in (0,1]$$ with

5 $$\begin{aligned} \psi (u,s)= \max \{d^{*}(u,s),d^{*}(u,[Tu]_{\alpha _{Tu}}),d^{*}(s,[Ls]_{\alpha _{Ls}}),\frac{d^{*}(u,[Ls]_{\alpha _{Ls}})+d^{*}(s,[Tu]_{\alpha _{Tu}})}{2y}\}. \end{aligned}$$

### **Theorem 3.2**

Let $(S,d^{*},y)$ be a *b*-metric space where $y\ge 1.$ Suppose there exists a continuous function $\texttt {F} \in \textsf {F}^*$ from the right. Let $L,T:S\rightarrow S^*$ be two fuzzy mappings satisfying $\texttt {F}$-contraction of Nadler type such that for all $s, u \in S$ $[Lu]_{\alpha _{Lu}},[Ts]_{\alpha _{Ts}}\in S^{CB}$. Then *L*, *T* have a common fuzzy fixed point. If $[Lu]_{\alpha _{Lu}} \ and \ [Ts]_{\alpha _{Ts}}$ are singleton subsets of *S* for all $s, u \in S$, then the common fuzzy fixed point of *T* and *L* is unique.

### *Proof*

Fix any $s \in S.$ Define $s_{0}=s$ and suppose $s_{1} \in [Ts_{0}]_{\alpha _{Ts_{0}}}$. By Lemma 2.22, there is $s_{2} \in [Ls_{1}]_{\alpha _{Ls_{1}}}$ and there exists $g>1$, so that

$$\begin{aligned} d^{*}(s_{1},s_{2})\le gH([Ts_{0}]_{\alpha _{Ts_{0}}},[Ls_{1}]_{\alpha _{Ls_{1}}}). \end{aligned}$$

By multiplying both sides by *y*, we get

$$\begin{aligned} yd^{*}(s_{1},s_{2})\le ygH([Ts_{0}]_{\alpha _{Ts_{0}}},[Ls_{1}]_{\alpha _{Ls_{1}}}), \end{aligned}$$

$$\Rightarrow $$

6 $$\begin{aligned} \texttt {F}(yd^{*}(s_{1},s_{2}))\le \texttt {F}(ygH([Ts_{0}]_{\alpha _{Ts_{0}}},[Ls_{1}]_{\alpha _{Ls_{1}}})). \end{aligned}$$

The continuity from the right of $\texttt {F} \in \textsf {F}^*$ yields that there is $g>1$ so that

7 $$\begin{aligned} \texttt {F}(gyH([Ts_{0}]_{\alpha _{Ts_{0}}},[Ls_{1}]_{\alpha _{Ls_{1}}}))\le \texttt {F}(yH([Ts_{0}]_{\alpha _{Ts_{0}}},[Ls_{1}]_{\alpha _{Ls_{1}}}))+a. \end{aligned}$$

From (6) and (7) we have

$$\begin{aligned} \texttt {F}(yd^{*}(s_{1},s_{2}))\le \texttt {F}(ygH([Ts_{0}]_{\alpha _{Ts_{0}}},[Ls_{1}]_{\alpha _{Ls_{1}}}))\le \texttt {F}(yH([Ts_{0}]_{\alpha _{Ts_{0}}},[Ls_{1}]_{\alpha _{Ls_{1}}}))+a. \end{aligned}$$

By Adding *a* on both sides and using Eq. (4), we get

$$\begin{aligned} a+ \texttt {F}(yd^{*}(s_{1},s_{2}))\le \texttt {F}(\beta \psi (s_{1},s_{2})). \end{aligned}$$

By using this iterating procedure, we build a sequence $\{s_{n}\}$ in *S* so that $s_{2n+1} \in [Ts_{2n}]_{\alpha _{Ts_{2n}}}$, $s_{2n+2} \in [Ls_{2n+1}]_{\alpha _{Ls_{2n+1}}}$ and

8 $$\begin{aligned} a+ \texttt {F}(yd^{*}(s_{2n+1},s_{2n+2}))\le \texttt {F}(\beta \psi (s_{2n},s_{2n+1})). \end{aligned}$$

The function F is strictly increasing. We obtain

$$\begin{aligned} (yd^{*}(s_{2n+1},s_{2n+2}))\le (\beta \psi (s_{2n},s_{2n+1})). \end{aligned}$$

That is,

9 $$\begin{aligned} d^{*}(s_{2n+1},s_{2n+2}))\le \psi (s_{2n},s_{2n+1}). \end{aligned}$$

Now, by using Lemma 2.18, we have

$$\begin{aligned} \psi (s_{2n},s_{2n+1})= & {} \max \{d^{*}(s_{2n},s_{2n+1}),d^{*}(s_{2n},[Ts_{2n}]_{\alpha _{Ts_{2n}}}),d^{*}(s_{2n+1},[Ls_{2n+1}]_{\alpha _{Ls_{2n+1}}}),\\{} & {} \frac{d^{*}(s_{2n},[Ls_{2n+1}]_{\alpha _{Ls_{2n+1}}})+d^{*}(s_{2n+1},[Ts_{2n}]_{\alpha _{Ts_{2n}}})}{2y}\}\\\le & {} \max \{d^{*}(s_{2n},s_{2n+1}),d^{*}(s_{2n},s_{2n+1}),d^{*}(s_{2n+1},s_{2n+2}),\frac{d^{*}(s_{2n},s_{2n+2})}{2y}\} \\= & {} \max \{d^{*}(s_{2n},s_{2n+1}),d^{*}(s_{2n+1},s_{2n+2}),\frac{d^{*}(s_{2n},s_{2n+2})}{2y}\} \\\le & {} \max \{d^{*}(s_{2n},s_{2n+1}),d^{*}(s_{2n+1},s_{2n+2}),\frac{y(d^{*}(s_{2n},s_{2n+1})+d^{*}(s_{2n+1},s_{2n+2}))}{2y}\}\\= & {} \max \{d^{*}(s_{2n},s_{2n+1}),d^{*}(s_{2n+1},s_{2n+2})\}. \end{aligned}$$

Suppose $$ d^{*}(s_{2n},s_{2n+1})< d^{*}(s_{2n+1},s_{2n+2})$$ then

$$\begin{aligned} \psi (s_{2n},s_{2n+1})< d^{*}(s_{2n+1},s_{2n+2}). \end{aligned}$$

It contradicts (9). Therefore, Eq. (8) implies that

10 $$\begin{aligned} a+ \texttt {F}(yd^{*}(s_{2n+1},s_{2n+2}))\le \texttt {F}(d^{*}(s_{2n},s_{2n+1})). \end{aligned}$$

Let $P_{n}=d^{*}(s_{2n+1},s_{2n+2})> 0$ for all $n \in \mathbb {N}.$ It follow from (10) that

11 $$\begin{aligned} a+ \texttt {F}(y^{n}d^{*}(s_{2n+1},s_{2n+2}))\le \texttt {F}(y^{n-1}d^{*}(s_{2n},s_{2n+1})) \,\, \forall \,\, n \in \mathbb {N}. \end{aligned}$$

Using Eq. (11), we write

12 $$\begin{aligned} \texttt {F}(y^{n}P_{n})\le & {} \texttt {F}(y^{n-1}P_{n-1})-a, \nonumber \\ \texttt {F}(y^{n-1}P_{n-1})\le & {} \texttt {F}(y^{n-2}P_{n-2})-2a, \nonumber \\&.&\nonumber \\&.&\nonumber \\&.&\nonumber \\ \texttt {F}(y^{n}P_{n})\le & {} \texttt {F}(y^{0}P_{0})-na. \end{aligned}$$

It yields

$$\begin{aligned} \lim _{n\rightarrow \infty }{} \texttt {F}(y^{n}P_{n})=-\infty . \end{aligned}$$

Using $\texttt {F}_{2}$ property, we have

$$\begin{aligned} \lim _{n\rightarrow \infty }(y^{n}P_{n})=0. \end{aligned}$$

Using $\texttt {F}_{3}$ property, there is $0 k<1$ such that

$$\begin{aligned} \lim _{n\rightarrow \infty }(y^{n}P_{n})^{k}{} \texttt {F}(y^{n}P_{n})=0. \end{aligned}$$

By inequality (12), we find that

13 $$\begin{aligned} \texttt {F}(y^{n}P_{n})\le \texttt {F} (y^{0}P_{0}) - na. \end{aligned}$$

By multiplying (13) by $(y^{n}P_{n})^{k}$, we obtain

$$\begin{aligned} (y^{n}P_{n})^{k}{} \texttt {F}(y^{n}P_{n})\le (y^{n}P_{n})^{k}{} \texttt {F}(P_{0})-na(y^{n}P_{n})^{k}. \end{aligned}$$

That is,

$$\begin{aligned} (y^{n}P_{n})^{k}{} \texttt {F}(y^{n}P_{n}) - (y^{n}P_{n})^{k}{} \texttt {F}(P_{0})\le -na(y^{n}P_{n})^{k} \le 0. \end{aligned}$$

Now, applying $\lim n\rightarrow \infty$, we get

14 $$\begin{aligned} \lim _{n\rightarrow \infty } n(y^{n}P_{n})^{k}=0. \end{aligned}$$

From (14), there is $n_{1} \in \mathbb {N}$ with $n(y^{n}P_{n})^{k}< 1$ such that

15 $$\begin{aligned} y^{n}P_{n}\le \frac{1}{n^{\frac{1}{k}}} \,\,\, \forall \, n\ge n_{1}. \end{aligned}$$

We claim that $\{s_{n}\}$ is a Cauchy sequence. Suppose $m,n \in \mathbb {N}$ are so that $m>n>n_{1}$. The triangular inequality and Eq. (15) both implies that

$$\begin{aligned} d^{*}(s_{2n},s_{2m})\le & {} y d^{*}(s_{2n},s_{2n+1})+ y^{2} d^{*}(s_{2n-1},s_{2n+2})+... + y^{m-n} d^{*}(s_{2m-1},s_{2m})\\= & {} yP_{n-1}+y^{2}P_{n}+...+y^{m-n}P_{m-2} \\= & {} \Sigma ^{m-2}_{i=n-1}y^{i-n+2}P_{i} \\\le & {} \Sigma ^{\infty }_{i=n-1}y^{i-n+2}P_{i} \\\le & {} y^{2-n}{\frac{1}{i^{\frac{1}{k}}}}. \end{aligned}$$

At the limit, we have $d^{*}(s_{n}, s_{m})\rightarrow 0.$ Hence, $\{s_{n}\}$ is a Cauchy sequence. The completeness of the *b*-metric space $(S,d^{*},y)$ ensures the existence of $s \in S$ so that $s_{n}\rightarrow s$ as $n \rightarrow \infty .$ Now, we show that *s* is a common fuzzy fixed point of the mappings *T* and *L*. Consider

$$\begin{aligned} d^{*}(s_{2n+2},[Ls]_{\alpha _{Ls}}) \le H([Ts_{2n+1}]_{\alpha _{Ts_{2n+1}}},[Ls]_{\alpha _{Ls}})\le yH([Ts_{2n+1}]_{\alpha _{Ts_{2n+1}}},[Ls]_{\alpha _{Ls}}). \end{aligned}$$

It implies

$$\begin{aligned} d^{*}(s_{2n+2},[Ls]_{\alpha _{Ls}}) \le yH([Ts_{2n+1}]_{\alpha _{Ts_{2n+1}}},[Ls]_{\alpha _{Ls}}). \end{aligned}$$

Since $\texttt {F}$ is strictly increasing, we get

$$\begin{aligned} \texttt {F}(d^{*}(s_{2n+2},[Ls]_{\alpha _{Ls}})) \le \texttt {F} (yH([Ts_{2n+1}]_{\alpha _{Ts_{2n+1}}},[Ls]_{\alpha _{Ls}})). \end{aligned}$$

By adding 2*a* on both sides and by using Eq. (4), we get

$$\begin{aligned} 2a+\texttt {F} (d^{*}(s_{2n+2},[Ls]_{\alpha _{Ls}}))\le 2a + \texttt {F} (yH([Ts_{2n+1}]_{\alpha _{Ts_{2n+1}}},[Ls]_{\alpha _{Ls}}))\le \texttt {F}(\beta \psi (s_{2n+1},s)). \end{aligned}$$

Since $a \in \mathbb {R^{+}}$, we get

$$\begin{aligned} \texttt {F} (d^{*}(s_{2n+2},[Ls]_{\alpha _{Ls}}))\le \texttt {F}(\beta \psi (s_{2n+1},s)). \end{aligned}$$

Since $\texttt {F}$ is strictly increasing, one writes

$$\begin{aligned} d^{*}(s_{2n+2},[Ls]_{\alpha _{Ls}})\le \beta \psi (s_{2n+1},s). \end{aligned}$$

By applying limit $n \rightarrow \infty ,$ we obtain

$$\begin{aligned} d^{*}(s,[Ls]_{\alpha _{Ls}3})\le \beta \psi (s,s). \end{aligned}$$

That is, $d^{*}(s,[Ls]_{\alpha _{Ls}})=0.$ Hence $s \in [Ls]_{\alpha _{Ls}}.$ Similarly, we can show that $s \in [Ts]_{\alpha _{Ts}}.$ Hence, *s* is a common fuzzy fixed point of *T* and *L*. Suppose that $[Ts]_{\alpha _{Ts}}$ and $[Lu]_{\alpha _{u}}$ are singleton subsets of *S* for all $s,u \in S$. Let *r* and *s* be two common fuzzy fixed points of the mappings *T* and *L*,  then

$$\begin{aligned} \texttt {F} (d^{*}(r,s))\le & {} \texttt {F} (yH(r,[Ls]_{\alpha _{Ls}}))+2a \\= & {} \texttt {F} (yH([Tr]_{\alpha _{Tr}},[Ls]_{\alpha _{Ls}}))+2a \\\le & {} \texttt {F}(\beta \psi (r,s)) \\= & {} \texttt {F} ( \beta \max \{d^{*}(r,s),d^{*}(r,[Tr]_{\alpha _{Tr}}),d^{*}(s,[Ts]_{\alpha _{Ts}}),\frac{d^{*}(r,[Ls]_{\alpha _{Ls}})+d^{*}(s,[Tr]_{\alpha _{Tr}})}{2y}\}) \\\le & {} \texttt {F} ( \beta \max \{d^{*}(r,s),0,0, \frac{d^{*}(r,[Ls]_{\alpha _{Ls}})+d^{*}(s,[Tr]_{\alpha _{Tr}})}{2y}\}) \\\le & {} \texttt {F} ( \beta \{d^{*}(r,s). \end{aligned}$$

It yields that $d^{*}(r,s) \le \beta d^{*}(r,s) < d^{*}(r,s).$ Hence, $d^{*}(r,s)=0$, and so $r=s.$ $\square$

To validate and furnish our result, we provide a non-trivial example below:

### *Example 3.3*

Let $S=[0,1].$ Define $d^{*}:S\times S \rightarrow \mathbb {R^{+}}$ by $d^{*}(s,u)=|s-u|^{2}.$ Then $(S,d^{*},y)$ is a *b*-metric space. Consider $a \in \mathbb {R^{+}}$ and $L,T: S \rightarrow S^*,$ such that $Ts:S \rightarrow [0,1]$ and $Lu:S \rightarrow [0,1]$ are given as

$$\begin{aligned} T(s)(t)= \left\{ \begin{array}{cc} \frac{1}{4}, &{} 0 \le t \le \frac{s e^{-a}}{6}; \\ \frac{1}{6}, &{} \frac{s e^{-a}}{6}< t \le \frac{s}{3}; \\ \frac{1}{5}, &{} \frac{s}{3}< t < \frac{s}{2}; \\ 0, &{} \frac{s}{2} \le t \le 1. \end{array} \right. \end{aligned}$$

There is $\alpha _{Ts}= \frac{1}{4}$ such that $[Ts]_{\alpha _{Ts}}=[0,\frac{s}{6}e^{-a}]$. Also,

$$\begin{aligned} L(u)(t)= \left\{ \begin{array}{ll} \frac{1}{4}, &{} 0 \le t< \frac{u e^{-a}}{6}; \\ \frac{1}{2}, &{} t = \frac{u e^{-a}}{6}; \\ \frac{1}{6}, &{} \frac{u e^{-a}}{6}< t < \frac{u}{2}; \\ 0, &{} \frac{u}{2} \le t \le 1. \end{array} \right. \end{aligned}$$

There is $\alpha _{Lu}= \frac{1}{2}$ such that $[Lu]_{\alpha _{Lu}}=\{\frac{u}{6}e^{-a}\}$. We have

$$\begin{aligned} H([Ts]_{\alpha _{Ts}},[Lu]_{\alpha _{Lu}})= & {} \max \{ \sup _{s\in [Ts]_{\alpha _{Ts}}} (d^{*}(s,[Lu]_{\alpha _{Lu}})), \sup _{u\in [Lu]_{\alpha _{Lu}}} (d^{*}([Ts]_{\alpha _{Ts}},u))\} \\ H([Ts]_{\alpha _{Ts}},[Lu]_{\alpha _{Lu}})= & {} \max \{ |\frac{s}{6}e^{-a}-\frac{u}{6}e^{-a}|^{2}, |0-\frac{u}{6}e^{-a}|^{2}\} \\\le & {} \frac{1}{36}e^{-2a} \max \{ |s-u|^{2},|u-\frac{u}{6}|^{2}\} \\\le & {} \frac{1}{36}e^{-2a} \max \{ |s-u|^{2},|u-\frac{u}{6}e^{-a}|^{2}\} \\= & {} \frac{1}{36}e^{-2a} \max \{ d^{*}(s,u), d^{*}(u,[Lu]_{\alpha _{Lu}})\} \\\le & {} \frac{1}{36}e^{-2a} \psi (s,u). \end{aligned}$$

This implies that

$$\begin{aligned} 3 H([Ts]_{\alpha _{Ts}},[Lu]_{\alpha _{Lu}})\le \frac{1}{12}e^{-2a} \psi (s,u). \end{aligned}$$

By taking natural logarithm on both sides and then by considering $y=3, \, \beta =\frac{1}{12}$ and $\texttt {F}(r)=\ln (r),$ all axioms of Theorem 3.2 hold, therefore *T* and *L* have a common fuzzy fixed point, which is, $s=0.$

### **Corollary 3.1**

Assume a metric space $(S,d^{*})$. Suppose there exists a continuous function $\texttt {F} \in \textsf {F}^*$ from the right. Let $L,T:S\rightarrow S^*$ be two fuzzy mappings satisfying $\texttt {F}$-contraction of Nadler type such that for all $s, u \in S$ $[Lu]_{\alpha _{Lu}},[Ts]_{\alpha _{Ts}}\in S^{CB}$. Then *L*, *T* have a common fuzzy fixed point. If $[Lu]_{\alpha _{Lu}} \ and \ [Ts]_{\alpha _{Ts}}$ are singleton subsets of *S* for all $s, u \in S$, then the common fuzzy fixed point of *T* and *L* is unique.

### **Corollary 3.2**

Assume a *b*-metric space $(S,d^{*},y)$, where $y\ge 1.$ Suppose there is a continuous function $\texttt {F} \in \textsf {F}^*$ from the right. Let $T:S\rightarrow S^*$ be a fuzzy mapping satisfying $\texttt {F}$-contraction of Nadler type such that for all $s \in S$, $[Ts]_{\alpha _{Ts}}\in S^{CB}$. Then *T* has a fixed point. If $\ [Ts]_{\alpha _{Ts}}$ are singleton subsets of *S* for all $s \in S$, then the fixed point of *T* is unique.

## Applications

### Finding common fixed points of multi-valued mappings

Here, we find common fixed points for multi-valued mappings with the help of our obtained result.

#### **Theorem 4.1**

Assume a *b*-metric space $(S,d^{*},y)$, where $y\ge 1.$ Suppose there exists a continuous function $\texttt {F} \in \textsf {F}^*$ from the right. If $A, B:S\rightarrow S^{CB}$ are two multi-valued mappings satisfying $\texttt {F}$-contraction of Nadler type, then *A* and *B* have a common fixed point. Moreover, if *A* and *B* are singleton mappings, then the common fixed point is unique.

#### *Proof*

Consider two arbitrary mappings $P, Q: S\rightarrow (0, 1]$. Define two fuzzy mappings $T, L: S \rightarrow S^{*}$ as follows:

$$ T(s)(g)={\left\{ \begin{array}{ll} P(s), & \quad if \ \ \ g \in A(s) \\ 0, & \quad if \ \ g \notin As, \end{array}\right. } $$

and

$$ L(u)(g)= {\left\{ \begin{array}{ll} Q(u), & \quad if \ \ \ g \in B(u) \\ 0, & \quad if \ \ g \notin B(u). \end{array}\right. } $$

Then for $$s, u \in S$$,

$$\begin{aligned} {[}Ts]_{\alpha _{Ts}}=\left\{ g\in S: T(s)(g) = P(s)\right\} = A(s), \end{aligned}$$

and

$$\begin{aligned} {[}Lu]_{\alpha _{Lu}}=\left\{ g\in S: L(u)(g)= Q(u)\right\} = B(u). \end{aligned}$$

Now, since $H([Ts]_{\alpha _{Ts}}, [Lu]_{\alpha _{Lu}})= H(A(s), B(u))$, Theorem 3.2 can be applied to obtain a common fixed point of *A* and *B*. That is, there is $r\in S$ such that $r \in T(r)\cap L(r)$ $\square$

#### **Corollary 4.1**

Assume a metric space $(S,d^{*})$. Suppose there exists a continues function $\texttt {F} \in \textsf {F}^*$ from the right. If $A, B:S\rightarrow S^{CB}$ are two multi-valued mappings satisfying $\texttt {F}$-contraction of Nadler type, then $A \ and \ B$ have a common fixed point. Moreover, if *A* and *B* are singleton mappings, then the common fixed point is unique.

#### **Corollary 4.2**

Assume a b-metric space $(S,d^{*},y)$, where $y\ge 1.$ Suppose there exists a continues function $\texttt {F} \in \textsf {F}^*$ from the right. If $A:S\rightarrow S^{CB}$ is a multi-valued mapping satisfying $\texttt {F}$-contraction of Nadler type, then *A* has a fixed point. Moreover, if *A* is a singleton mapping, then fixed point of *A* is unique.

### Existence solution of a system of non-linear Fredholm integral equations of 2nd kind

In this section, we apply our obtained results to establish some hypothesis which guarantee the existence of solution of system of non-linear Fredholm integral equations of 2nd kind.

Consider the following system of Fredholm integral equations of 2nd kind:

16 $$\begin{aligned} {\left\{ \begin{array}{ll} u(t_{1})= \phi (t_{1})+ \int _{a}^{b}B_{1}(t_{1},y_{1},u(y_{1}))d^{*}y_{1},, t_{1}\in [a,b],\\ s(t_{1}) =\phi (t_{1})+ \int _{a}^{b}B_{2} (t_{1},y_{1},s(y_{1}))d^{*}y_{1},, t_{1}\in [a,b]. \end{array}\right. } \end{aligned}$$

We will present sufficient conditions to ensure the existence of solutions to such a system. Let $S=C[a,b]$ be the set of all continuous functions defined on [*a*, *b*]. Define $d^{*}:S \times S \rightarrow \mathbb {R^{+}}$ by

$$\begin{aligned} d^{*}(s,u)=\sup _{t_{1}\in [a,b]}|s(t_{1})-u(t_{1})|^{2}. \end{aligned}$$

Then $(S,d^{*},y)$ is a complete *b*-metric space on *S*.

#### **Theorem 4.2**

Assume the assumptions given below hold: $(A_{1})$ $B_{i} : [a,b] \times [a,b] \times \mathbb {R^{+}} \rightarrow \mathbb {R^{+}}$ (for $i=1,2$) and $\phi : [a, b] \rightarrow \mathbb {R^{+}}$ are continuous;

$(A_{2})$ There exists a continuous function $J:[a,b] \times [a,b] \rightarrow [0, \infty )$ such that

$$\begin{aligned} |B_{i}(t_{1},y_{1},v)|-|B_{j}(t_{1},y_{1},w)| \le J(t_{1},y_{1})|v-w| \end{aligned}$$

for each $t_{1}, y_{1} \in [a,b];$ $(A_{3})$ $\sup _{t_{1},y_{1} \in [a,b]} \int _{a}^{b}|J(t_{1},y_{1})|d^{*}y_{1} \le \sqrt{\frac{\beta }{y}.e^{-a}}$, where $0<\beta <1.$ Then the system of integral equations (16) has a common solution in *C*([*a*, *b*]).

#### *Proof*

Let $\omega$ and $\theta$ be two self-mappings $\omega , \theta : C([a,b]) \rightarrow C([a,b])$ defined by

$$\begin{aligned} \omega (u(t_{1}))= \phi (t_{1})+ \int _{a}^{b}B_{1}(t_{1},y_{1},u(y_{1}))d^{*}y_{1}, \, t_{1}\in [a,b], \\ \theta (s(t_{1})) =\phi (t_{1})+ \int _{a}^{b}B_{2} (t_{1},y_{1},s(y_{1}))d^{*}y_{1}, \, t_{1}\in [a,b]. \end{aligned}$$

Consider two arbitrary mappings $A, B: S\rightarrow (0, 1]$.

Define two fuzzy mappings $T, L: S \rightarrow S^{*}$ as follows:

$$\begin{aligned} T(s)(g)=\left\{ \begin{array}{ll} A(s), \ \ if \ \ \ g(t_{1})=\theta (s(t_{1})); \ \forall t_{1} \in [a,b] \\ 0, \ \ \ \ \ \ otherwise. \end{array} \right. \\ L(u)(g)=\left\{ \begin{array}{ll} B(u), \ \ if \ \ \ g(t_{1})=\omega (u(t_{1})); \ \forall t_{1} \in [a,b] \\ 0, \ \ \ \ \ \ otherwise. \end{array} \right. \end{aligned}$$

Take $\alpha _{Ts}=A(s)$ and $\alpha _{Lu}=B(u)$, then

$$\begin{aligned}{}[Ts]_{\alpha _{Ts}}=\left\{ g\in S: T(s)(g) = A(s)\right\} =\theta (s), \end{aligned}$$

and

$$\begin{aligned} {[}Lu]_{\alpha _{Lu}}=\left\{ g\in S: L(u)(g)= B(u)\right\} =\omega (u). \end{aligned}$$

We have

$$\begin{aligned} H([Lu]_{\alpha _{Lu}},[Ts]_{\alpha _{Ts}})= & {} d^{*}(\omega (u), \theta (s)) \\= & {} \sup _{t_{1} \in [a,b]}|\omega (u)(t_{1})-\theta (s)(t_{1})|^{2} \\\le & {} \sup _{t_{1} \in [a,b]}(\int _{a}^{b}|B_{1}(t_{1},y_{1},v)|-|B_{2}(t_{1},y_{1},w)|)^{2} \\\le & {} \sup _{t_{1} \in [a,b]}(\int _{a}^{b}J(t_{1},y_{1})|u(t_{1})-s(t_{1})|d^{*}y_{1})^{2} \\\le & {} \sup _{t_{1} \in [a,b]}(\int _{a}^{b}|u(t_{1})-s(t_{1})|d^{*}y_{1})^{2}\sup _{t_{1} \in [a,b]}(\int _{a}^{b}J(t_{1},y_{1})d^{*}y_{1})^{2} \\\le & {} (\sup _{t_{1} \in [a,b]}|u(t_{1})-s(t_{1})|)^{2} \frac{\beta e^{-2a}}{y} \\= & {} \frac{\beta e^{-2a}}{y}d^{*}(u,s). \end{aligned}$$

That is,

$$\begin{aligned} H([Lu]_{\alpha _{Lu}},[Ts]_{\alpha _{Ts}}) \le \frac{\beta e^{-2a}}{y}d^{*}(u,s) \le \frac{\beta e^{-2a}}{y}\psi (u,s). \\ yH([Lu]_{\alpha _{Lu}},[Ts]_{\alpha _{Ts}}) \le e^{-2a} \beta \psi (u,s). \end{aligned}$$

By taking $F(r)= \ln (r)$ in Theorem 3.2, the system of integral equations (16) has a common solution. $\square$

## Conclusion

Fixed point theory is a useful theoretical tool in diverse fields, such as logic programming, functional analysis and artificial intelligence. In the framework of b-metric spaces, a novel fuzzy fixed point result of two fuzzy mappings satisfying F-contraction is established in connection with Housdorff metric. Obtained result is furnished with an interesting and non-trivial example. Some results for fuzzy mappings and multi-valued mappings are incorporated as corollaries. Moreover, other direct consequences are obtained as well. Moreover, a system of non-linear Fredholm integral equations is solved by our established result. We hope this existence result will provide an appropriate environment to approximate further operator equations in applied science. We conclude our work with some open questions:

1. Whether this type of contraction can be applied on more than two mappings?
2. If answer to 1 is yes then is this give the surety of existence of coincidence points or common fixed points?
3. Whether these results can be obtained in other generalizations of metric spaces?

## Data Availability

The database used and analysed during the current study are available from the corresponding author on reasonable request.
